# Clinical manifestations of drug reaction with eosinophilia and systemic symptoms in pediatric patients

**DOI:** 10.1016/j.jdcr.2024.06.041

**Published:** 2024-08-27

**Authors:** Suzanne Blackley, Chantal Lemoine-Soto, Li Zhou, Adaeze Ezeofor, Ying-Chih Lo, Kimberly G. Blumenthal, Daniela Kroshinsky

**Affiliations:** a Division of General Internal Medicine, Department of Medicine, Brigham and Women’s Hospital, Boston, Massachusetts; b Division of Rheumatology, Allergy, and Immunology, Department of Medicine, Massachusetts General Hospital, Boston, Massachusetts; c Harvard Medical School, Boston, Massachusetts; d Department of Medicine, Massachusetts General Hospital, Boston, Massachusetts; e The Mongan Institute, Massachusetts General Hospital, Boston, Massachusetts; f Department of Dermatology, Duke University Medical Center, Durham, North Carolina

**Keywords:** children, DIHS, drug allergy, hypersensitivity, SCAR, severe cutaneous adverse reaction

Drug reaction with eosinophilia and systemic symptoms (DRESS) can be difficult to diagnose in children due to its rarer occurrence, tendency to mimic other common pediatric conditions, and limited scientific studies.^1^, ^2^, ^3^ This study describes a series of pediatric DRESS cases and compares their clinical manifestations to those of adult DRESS cases at a large academic medical center (Fig 1).

**Fig 1:**
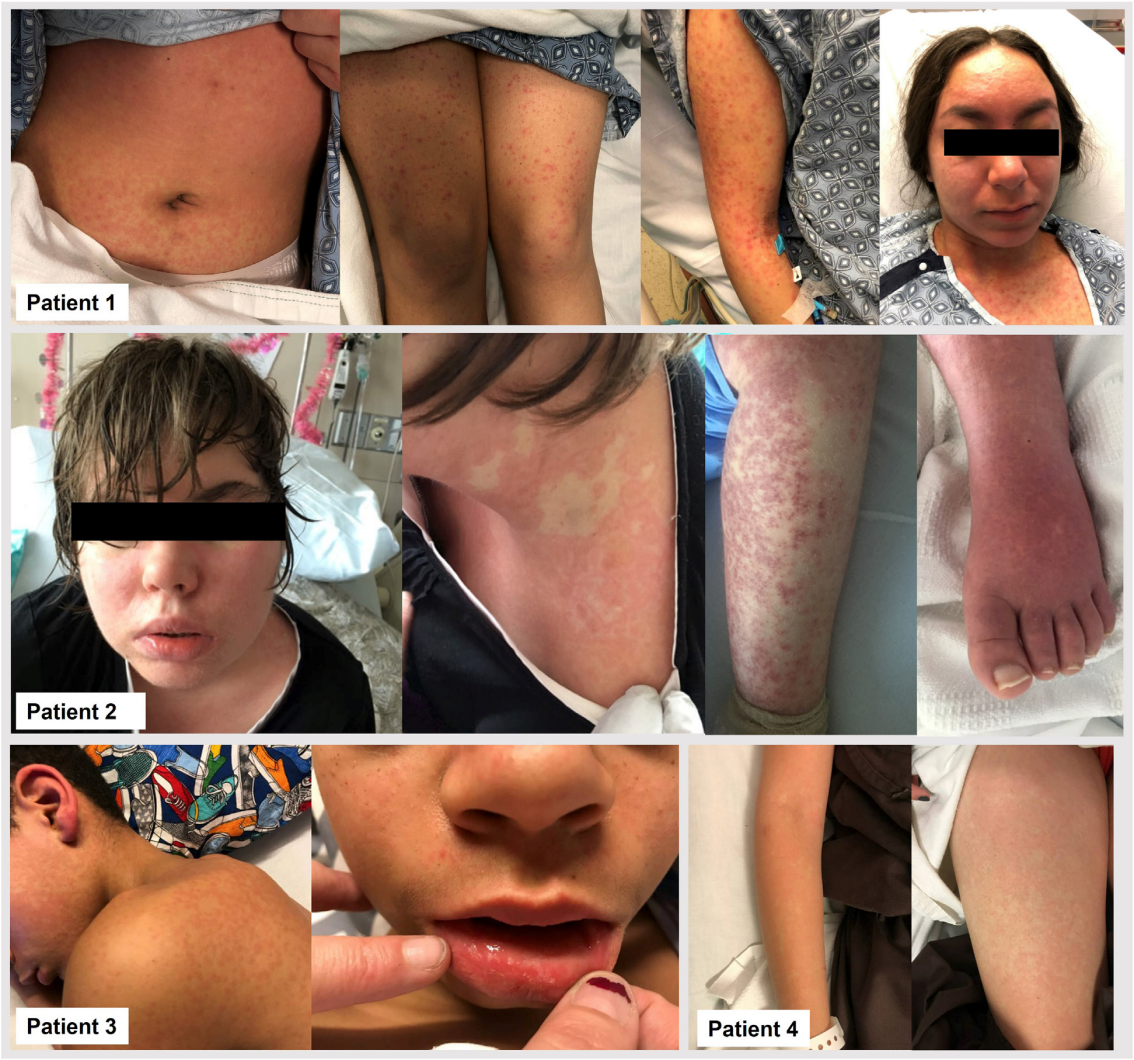
Cutaneous manifestations of DRESS in the study population.

This retrospective chart review leverages 30 years of longitudinal electronic health record data within the Mass General Brigham healthcare system. Specialist-diagnosed DRESS cases were identified using natural language processing on multiple electronic health record data types.^4^ Cases were validated through manual chart review. Clinical documentation was used to obtain Registry of Severe Cutaneous Adverse Reaction (RegiSCAR) scores of each DRESS case.^5^ Clinical features, culprit drug, and treatment outcomes, including length of hospital stay (LOS) and deaths attributed to DRESS, were assessed.

Of 7 pediatric cases, the median age was 15 years [range: 9-17 years; IQR: 14; 16 years; Table I]. RegiSCAR scores indicated “probable” (*n* = 4, 57%), “possible” (*n* = 3, 43%), with no pediatric cases considered “definite.” Mean RegiSCAR score was 3.6 (SD: 0.9). All pediatric cases presented with fever and morbilliform rash. Common systemic manifestations included liver injury (*n* = 5, 71%), lymphadenopathy (*n* = 4, 57%), and atypical lymphocytes (*n* = 2, 29%). Eosinophilia was present in 4 (57%) of cases with an average peak absolute eosinophilia count of 1000/L (SD: 2000/L). Culprit agents were vancomycin (*n* = 3, 43%), antiepileptics (*n* = 2, 29%), minocycline (*n* = 1, 14%), and ibuprofen (*n* = 1, 14%). Median LOS for acute DRESS was 6 days [range: 2-27 days; IQR: 6, 14 days], and there were no deaths attributed to DRESS.

**Table I:** Clinical outcomes of drug reaction with eosinophilia and systemic symptoms in pediatric compared to adult patients

*N* (%) unless specified	Adult (age 18+) *n* = 253	Pediatric (age <18) *n* = 7
Demographics		
Age, median [range; IQR]	55 [18-97; 45, 66]	15 [9-17; 14, 16]
Sex, female	135 (53)	6 (86)
Race/ethnicity		
White	180 (71)	3 (43)
Black	28 (11)	1 (14)
Asian	18 (7)	0 (0)
Other or missing	27 (11)	3 (43)
Comorbidities∗		
Hypertension	103 (41)	0 (0)
Diabetes	45 (18)	0 (0)
Malignant disease/cancer	44 (17)	0 (0)
Coronary artery disease	26 (10)	0 (0)
Congestive heart failure	25 (10)	0 (0)
RegiSCAR		
>5 (definite)	55 (22)	0 (0)
4-5 (probable)	101 (40)	4 (57)
2-3 (possible)	97 (38)	3 (43)
Mean RegiSCAR score	4.2 ± 1.7	3.6 ± 0.9
Skin rash		
Onset (days from suspected drug initiation)	10 ± 15	16 ± 9
Rash extent (skin surface %)	64 ± 22	66 ± 11
Rash description†		
Erythema	188 (74)	5 (71)
Morbilliform	81 (32)	2 (29)
Erythroderma	60 (24)	2 (29)
Macules	47 (19)	2 (29)
Systemic features		
Eosinophilia‡	192 (76)	4 (57)
Fever ≥ 100.6 F	189 (75)	7 (100)
Liver injury§	156 (62)	5 (71)
Kidney injury‖	114 (45)	1 (14)
Atypical lymphocytes	82 (32)	2 (29)
Involved mucous membranes¶	55 (22)	0 (0)
Lymphadenopathy#	45 (18)	4 (57)
Peak AEC/L∗∗	2000 ± 3000	1000 ± 2000
Causal drugs		
Vancomycin	88 (35)	2 (29)
Cephalosporin	51 (20)	0 (0)
Antiepileptic	33 (13)	2 (29)
TMP-SMX††	31 (12)	0 (0)
Allopurinol	14 (6)	0 (0)
Other and unidentified	89 (35)	1 (14)
Treatment(s)‡‡		
Steroids (IV and/or oral)	196 (78)	6 (86)
Topical steroids	133 (53)	4 (57)
Antihistamine	106 (42)	4 (57)
Cyclosporine	11 (4)	1 (14)
Epinephrine	4 (2)	0 (0)
IVIG§§	1 (0)	1 (14)
Hospitalized for DRESS		
Hospitalization	146 (58)	5 (71)
Outcomes		
Length of hospital stay, median (range, IQR)	11 [range: 0-165 d, IQR: 6, 26 d]	6 d [2-27; 6, 14]
In-hospital mortality	14 (6)	0 (0)
Death attributed to DRESS	3 (1)	0 (0)

∗ Displaying only those present in ≥10% of the adult population.

† Displaying only those present in ≥10% of the population.

‡ Eosinophilia defined as absolute eosinophil count (AEC) > 500/L.

§ Liver injury defined as alanine transaminase (ALT) ≥ 100 U/L, elevated liver function or transaminitis.

‖ Kidney injury defined as creatinine increase by 0.5 mg/dL or 50% above baseline.

¶ Involved mucous membranes include ocular, oral, genital, other, or none.

# Lymphadenopathy defined as at least 2 sites with lymph nodes 1 cm or greater.

∗∗ AEC, absolute eosinophil count.

†† TMP-SMX, trimethoprim/sulfamethoxazole.

‡‡ Multiple treatments were commonly combined in clinical practice.

§§ IVIG, intravenous immune globulin.

Of 253 adult cases, the median age was 55 years [range: 18-97 years; IQR: 45, 66 years]. RegiSCAR scores indicated “definite” (*n* = 55, 22%), “probable” (*n* = 101, 40%), and “possible” (*n* = 97, 38%). Mean RegiSCAR score was 4.2 (SD: 1.7). Common clinical manifestations included fever (*n* = 189, 75%) and rash (*n* = 248, *n* = 98%) that was commonly characterized as macular, morbilliform, or maculopapular (*n* = 221, 87%). Common systemic manifestations included liver injury (*n* = 156, 62%), kidney injury (*n* = 114, 45%), and atypical lymphocytes (*n* = 82, 32%); lymphadenopathy (*n* = 45, 18%) was less prevalent. Eosinophilia was more prevalent (*n* = 192, 76%), with an average peak eosinophilia count of 2000/L (SD: 3000/L). Culprit agents were vancomycin (*n* = 88, 35%), cephalosporins (*n* = 51, 20%), antiepileptics (*n* = 33, 13%), trimethoprim-sulfamethoxazole (*n* = 31, 12%), and allopurinol (*n* = 14, 6%). Median LOS for acute DRESS was 11 days (range: 0-165 days; IQR: 6, 26 days), although 107 (42%) were hospitalized for a condition other than DRESS; there were 3 deaths attributed to DRESS (1.2%).

In this study with 7 pediatric cases, compared to the 253 adult cases from the same health care system, DRESS tended to impact female older adolescents and middle-aged adults. Vancomycin was the most common culprit agent across all ages. While cephalosporins were the second most common culprit agents in adults, antiepileptics were the second most common culprit agents in children. This is consistent with current literature citing antibiotics such as vancomycin along with antiepileptics as commonly identified DRESS culprits.^2^^,^^3^^,^^6^

When organ injury was present, liver injury was most prevalent in children compared to both liver and kidney injury being prevalent in adults. Lymphadenopathy was more frequently recognized and/or documented in pediatric DRESS cases. Mean peak absolute eosinophil counts were twice as high in adults than in pediatric patients.

DRESS tended to be milder in pediatric patients, with a shorter LOS and no DRESS-attributed deaths. However, these outcomes may be confounded given the broad array of medical comorbidities present in adults who were also largely hospitalized for reasons other than DRESS.

A greater incongruence between the clinical DRESS diagnosis and RegiSCAR score existed in pediatric cases. More pediatric cases scored “probable” or “possible” and fewer scored “definite,” potentially suggesting that DRESS continues to be more difficult to diagnose in children.^2^ As there are few studies on the application of RegiSCAR scoring criteria in children, new scoring systems may need to be developed for the pediatric population.

Our small pediatric sample size limits the power to compare DRESS in children and adults. Large-scale studies of DRESS cases, including in pediatrics, are needed.

## Conflicts of interest

Dr Blumenthal receives grant support from the NIH/NIAID (R01AI150295), Phadia Ab (Thermo Fisher Scientific), and the Massachusetts General Hospital; personal fees for legal case review from Weekly Schulte Valdes Murman Tonelli, Piedmont Liability Trust, Vasios Kelly and Stollo PA, and Publix Supermarkets; and royalties from UpToDate, outside the submitted work. Drs Blackley, Lemoine-Soto, Zhou, Ezeofor, Lo, and Kroshinsky have no conflicts of interest to declare.
